# PI3K/AKT/mTOR Signaling Pathway in Breast Cancer: From Molecular Landscape to Clinical Aspects

**DOI:** 10.3390/ijms22010173

**Published:** 2020-12-26

**Authors:** Daniela Miricescu, Alexandra Totan, Iulia-Ioana Stanescu-Spinu, Silviu Constantin Badoiu, Constantin Stefani, Maria Greabu

**Affiliations:** 1 Department of Biochemistry, Faculty of Dental Medicine, Carol Davila University of Medicine and Pharmacy, 8 Eroii Sanitari Blvd, 050474 Bucharest, Romania; daniela.miricescu@umfcd.ro (D.M.); alexandra.totan@umfcd.ro (A.T.); maria.greabu@umfcd.ro (M.G.); 2 Department of Anatomy and Embryology, Faculty of Medicine, Carol Davila University of Medicine and Pharmacy, 8 Eroii Sanitari Blvd, 050474 Bucharest, Romania; 3 Department of Family Medicine and Clinical Base, Dr. Carol Davila Central Military Emergency University Hospital, 134 Calea Plevnei, 010825 Bucharest, Romania; constantin.stefani@umfcd.ro

**Keywords:** breast cancer, estrogen receptor-positive, HER2, PI3K/AKT/mTOR pathway, endocrine resistance

## Abstract

Breast cancer is a serious health problem worldwide, representing the second cause of death through malignancies among women in developed countries. Population, endogenous and exogenous hormones, and physiological, genetic and breast-related factors are involved in breast cancer pathogenesis. The phosphatidylinositol 3-kinase (PI3K)/protein kinase B (AKT)/mammalian target of rapamycin (mTOR) is a signaling pathway involved in cell proliferation, survival, invasion, migration, apoptosis, glucose metabolism and DNA repair. In breast tumors, PIK3CA somatic mutations have been reported, located in exon 9 and exon 20. Up to 40% of PIK3CA mutations are estrogen receptor (ER) positive and human epidermal growth factor receptor 2 (HER2) -negative in primary and metastatic breast cancer. HER2 is overexpressed in 20–30% of breast cancers. HER1, HER2, HER3 and HER4 are membrane receptor tyrosine kinases involved in HER signaling to which various ligands can be attached, leading to PI3K/AKT activation. Currently, clinical studies evaluate inhibitors of the PI3K/AKT/mTOR axis. The main purpose of this review is to present general aspects of breast cancer, the components of the AKT signaling pathway, the factors that activate this protein kinase B, PI3K/AKT-breast cancer mutations, PI3K/AKT/mTOR-inhibitors, and the relationship between everolimus, temsirolimus and endocrine therapy.

## 1. Breast Cancer–Incidence and Risk Factors

Breast cancer is a serious medical condition which especially affects women, but also men, in the Unites States of America (USA) 1% of the total cases of breast malignant tumors being diagnosed in male patients [1]. Breast cancer is the main type of carcinoma in women [2,3] and the second most common type of neoplasia in the general population, in the world [4]. Almost 270,000 women are newly diagnosed with breast cancer every year in the USA [5,6,7]. In developed countries, it is the second cause of death through malignancies in women after lung cancer [5,6,7]. About 42,000 women died in the USA as a consequence of breast cancer in 2019 [5,6,7]. The lifetime risk of dying of breast cancer is approximately 2.6% [7]. The lifetime risk of developing breast cancer in the United States of America is 12.4% [8]. The incidence of this pathology varies with race and ethnicity [9]. Breast cancer incidence also presents variations related to the geographic zone, ranging from 27/100,000 (Central-East Asia and Africa) to 85–94/100,000 (Australia, North America and Western Europe) [4,10]. The mortality rate varies from six cases per 100,000 people in East Asia to 20 cases per 100,000 people in Western Africa [4]. Although the incidence of breast cancer is higher in developed countries, the mortality is higher in low-income countries because of late diagnosis, lack of screening and poor access to treatment [11]. Many factors influencing breast cancer have been studied. Age is the most prominent risk factor for breast cancer, after gender [12]. The incidence rate of breast cancer increases with age and reaches a peak around the age of 50. Age of menarche and age of menopause can also influence breast cancer development, given the effects of the ovarian hormones on the mammary gland from puberty till menopause. Early menarche, time of menopause onset and oophorectomy can play an important role in breast cancer development as well [12,13,14,15].

Regarding gender prevalence, it is common knowledge that breast cancer is much more frequent in women than men [16]. Because it is so rare in men, breast cancer is underdiagnosed and, in many cases, the diagnostic is obtained in more advanced stages than in women. A controversial risk factor for breast cancer is represented by the blood group; women with blood group A and Rh positive present a higher risk for breast cancer than women with blood group AB and Rh negative [17]. In addition, studies have shown that black women have a higher incidence of breast cancer than white women before the age of 40. After 40 years of age, the situation reverses [18]. Asian, Hispanic and Indian American women have a lower incidence rate of breast cancer than white and black American women [19].

Full-term pregnancy is a protective factor against breast cancer [20,21,22], with nulliparous women having an increased risk for breast cancer [23]. On the other hand, during the first pregnancy, important and permanent changes in the mammary gland cells occur: the glandular cells have a longer cell cycle and a prolonged G1 phase (Gap 1 phase)—it is the phase that allows DNA to repair. Consequently, the risk of the appearance of DNA alterations (that will be transmitted with the proliferation of breast gland cells during pregnancy) increases with the age of the women at first full term pregnancy [24,25,26]. Additionally, more than one birth and more closely spaced births are considered to be protective factors against breast cancer [24].

Breastfeeding is a protective factor against breast cancer, the longer the breastfeeding, the greater the protective effect [27,28]. At the same time, the association of two protective factors (two or more childbirths and lactation for more than 13 months) could reduce the risk of developing breast cancer by up to 60% [29,30].

In postmenopausal women, the risk of breast cancer increases proportionally with estrogen levels, with the use of antiestrogens (tamoxifen, raloxifene) proving to be effective in preventing the development of breast cancer [31,32]. Testosterone can also promote breast cancer development in postmenopausal women [31], acting by its conversion to estrogen and by its anabolic effect upon breast cancer cells (apparently for receptor-positive breast cancers) [31,33].

Although clinical and epidemiological studies have controversial results, prolactin, insulin-like growth factor (IGF-1) and oral contraceptives used longer than 10 years have all been incriminated as risk factors for breast cancer [34,35,36,37,38,39,40]. Additionally, hormone replacement therapy can enhance the risk of developing breast cancer and amplify the rate of mortality. The risk decreases after a minimum of 5 years of discontinuation of the treatment [41,42]. Moreover, the increased risk of developing breast cancer varies, pending to the hormones used, while the association of progesterone determines a greater risk for breast cancer [43,44,45]. The influence of ovulation stimulating drugs in breast cancer pathogenesis is still controversial, with studies reporting different results [46,47].

Family history of breast cancer and mutations of the genes codifying the synthesis of enzymes (matrix metalloproteinases, hormone-metabolizing enzymes), interferon alpha, estrogen and progesterone receptors or the genes involved in DNA repair represent major risk factors as well [48,49,50,51,52,53].

Diabetes mellitus (mainly type II) correlates with an increased risk of breast cancer, especially when associated with an elevated BMI (Body Mass Index) and with high levels of IGF-1 [54,55,56]. The use of metformin increases the survival rate in type II diabetic patients with breast cancer [57]. An increased BMI correlates with an increased risk of breast cancer [58,59], with obesity after menopause representing a proven risk factor for breast carcinoma [60,61]. Increased intake of meat and saturated fats [62,63] and the consumption of milk before menopause determine increased risk of breast cancer [64], while decreased serum levels of vitamin D are associated with a higher risk of breast cancer [65]. The administration of vitamin D supplements reduces the risk of breast cancer [66], but alcohol consumption constitutes a risk factor [67,68], as well as active and/or passive smoking [69,70], although there are studies that show different results [71].

On the other hand, physical activity reduces the risk of breast cancer [72,73]. However, working overnight leads to exposure to artificial light, increasing the level of estrogen and, consequently, augmenting the risk of breast cancer [74,75,76].

Women with higher social and economic status have an increased risk of breast cancer (older age at the first childbirth, older age at menopause, sedentary life, and unhealthy diet). However, they have more frequent medical examinations, hence can benefit from earlier diagnosis [77,78]. Meanwhile, women with lower social and economic status are later diagnosed and have a poorer prognosis [79].

Non-proliferative breast diseases, such as mild hyperplasia, cysts and apocrine metaplasia, do not increase the risk of breast cancer, and neither do breast implants [80,81]. Meanwhile, proliferative breast diseases without atypia (moderate hyperplasia, intraductal papilloma, sclerosing adenoma) increase the risk of breast cancer up to two-fold. Moreover, proliferative breast diseases with atypia (ductal hyperplasia with atypia and lobular hyperplasia with atypia) enhance the risk of developing this type of carcinoma up to six-fold [80]. Increased mammographic density is the most important risk factor for breast carcinoma after family history of breast cancer [82], with breast density being an independent risk factor [83,84]. Breast exposure to radiation by accident, therapeutically or for screening, can also increase the risk of malignancy [85,86,87].

## 2. Molecular Types of Breast Cancer

Using gene expression profiling, breast cancer molecular types were described [88]. In order to define the types of breast cancer, the expression of three tumor markers was studied:

1. ER-estrogen receptor status.
2. PR-progesterone receptor status.

Note that HR-represents the joint assessment of ER and PR status.

3. HER2-human epidermal growth factor receptor 2 status.

This is how the main molecular subtypes of breast cancer were defined:

1. HR+/HER2– corresponding to Luminal A subtype.
2. HR+/HER2+ corresponding to Luminal B subtype.
3. HR−/HER2+ corresponding to HER2 enriched subtype.
4. HR−/HER2– corresponding to triple negative subtype.

The most frequent is HR+/HER−, accounting for about 70% of breast cancers [89]. The prognosis is different for each molecular type. Luminal A breast cancer is hormone-receptor-positive (estrogen-receptor and/or progesterone-receptor-positive), HER2 negative, and has low levels of the protein Ki-67, which helps control how fast cancer cells grow. Luminal A cancers are low-grade, tend to grow slowly and have the best prognosis: 80–85% 5-year survival [90]. Luminal B breast cancer is hormone-receptor-positive (estrogen-receptor and/or progesterone-receptor-positive), and either HER2 positive or HER2 negative with high levels of Ki-67. The expression status of proliferation linked genes is one of the most important factors of the difference between luminal A and luminal B breast cancers. Luminal B cancers generally grow slightly faster than luminal A cancers and their prognosis is worse [90].

HER2-enriched breast cancer is hormone-receptor negative (estrogen-receptor and progesterone-receptor negative) and HER2 positive. HER2-enriched cancers tend to grow faster than luminal cancers and can have a worse prognosis: approximately 50–60% 5-year survival. They are often successfully treated with targeted therapies aimed at the HER2 protein [90]. Triple-negative/basal-like breast cancer is hormone-receptor negative (estrogen-receptor and progesterone-receptor negative) and HER2 negative, being more common in women with BRCA1 gene mutations. There is not a perfect match between basal-like breast cancer and triple-negative breast cancer [91]. Recently, seven different subtypes have been described for the triple-negative breast cancer based on analysis of gene expression profiles: basal-like 1 (BL1), basal-like 2 (BL2), immunomodulatory (IM), mesenchymal-like (M), mesenchymal stem–like (MSL), luminal androgen receptor (LAR), and unstable (UNS) [92]. The basal-like and HER2+ subtypes are more aggressive, having a higher proportion of major gene expression signatures [91]. Normal-like breast cancer is similar to luminal A disease: hormone-receptor-positive (estrogen-receptor and/or progesterone-receptor-positive), HER2 negative, and has low levels of the protein Ki-67, which helps control how fast cancer cells grow. Still, while normal-like breast cancer has a good prognosis, its outlook is slightly worse compared to luminal A cancer. Ki-67 is a nuclear antigen present in some phases of the cell cycle (mid G1, S, G2, and the entire M) that was found to be overexpressed in women with a shorter metastases-free survival period [91,93].

## 3. PI3K/AKT/mTOR Signaling Pathway

Cells intercommunicate in a process called extracellular signaling. They produce specific molecules that bind to specific receptors of other cells and activate intracellular signaling pathways. This is how cells respond to changes and adapt [94].

The PI3K/AKT/mTOR complex is a signaling pathway with a major role in essential cellular activities, such as: cell metabolism, cell growth, cell proliferation, apoptosis, and angiogenesis [94]. A ligand (for instance insulin or an insulin-like growth factor) binds to a cell-membrane receptor (such as receptors for tyrosine kinases or G-protein-coupled-receptors-GPCR). The specific receptor, activated by the extracellular ligand, activates PI3K (phosphatidylinositol (3,4,5)-trisphosphate kinase). The activated PI3K catalyzes phosphorylation of PIP2 at the 3 position of the inositol ring to generate PIP3, which recruits two protein kinases to the plasma membrane via their pleckstrin homology interaction domains (PH domains): AKT (also called protein kinase B, or PKB) and PDK1 (phosphoinositide-dependent protein kinase 1). Once recruited to the cell membrane, the AKT is phosphorylated by mTORC2 (mTOR complex 2) on Ser473, changing the conformation of the AKT and allowing its phosphorylation on Thr308 by PDK1. The activated AKT phosphorylates target proteins from the cell membrane, then loses its connection with the cell membrane and phosphorylates other target proteins in the cytosol and cell nucleus. The phosphorylation of target proteins results in the stimulation of cell survival, growth, and proliferation [94].

### 3.1. PI3K/AKT/mTOR Signaling Pathway Members

#### 3.1.1. PI3K-Phosphatidylinositol 3-Kinase-(Phosphoinositide 3-Kinase)

PI3K is a plasma-membrane-bound enzyme activated by RTKs (receptor tyrosine kinases) and by GPCRs (G protein-coupled receptors). GPCRs are the largest class of cellular surface receptors, with a generic structure; each GPCR is a transmembrane single polypeptide chain that uses G proteins to transmit the signal into the cytosol [94,95,96,97]. RTKs are a large family of plasma membrane receptors, too, with intrinsic protein kinase activity [95,96,97]. PI3Ks phosphorylate the 3’ position of the inositol head group of phosphatidylinositol (PIP2 and PIP3) lipids. PIP3 is the effector of multiple downstream targets of the phosphoinositide 3 kinase (PI3K) pathway [95,96,97]. There are many phosphatidylinositol 3-kinases-PI3Ks, divided into three groups or classes: PI3Ks class I, PI3Ks class II and PI3Ks class III [98].

PI3Ks class I is subdivided into PI3Ks class IA, PI3Ks class IB and PI3Ks class IC. IA-PI3Ks are heterodimers, consisting of a regulatory unit (p85α, p85β, p85γ) that activates the catalytic unit (p110α, p110β, p110δ, p110γ [98]). The IA-PI3Ks are activated directly by cell surface receptors: G protein-coupled receptors, RTKs and the small G protein RAS. Small GTPases form a superfamily within the larger class of regulatory GTP hydrolases, while RAS proteins are small GTPases that regulate cell growth, proliferation and differentiation [94,95,96,97]. IA-PI3Ks are present in many types of tissues and are activated by G protein- coupled receptors [98,99]. IB-PI3Ks are heterodimers containing the p101 regulatory subunit, which activates the p110γ catalytic subunit [99,100,101].

Class II PI3Ks has three isoforms: PI3KC2α and PI3KC2β are expressed in most of the tissues and organs, while PI3KC2γ is expressed only in the liver [102]. They regulate intracellular membrane dynamics and membrane traffic [102,103]. Class III PI3Ks has only one member identified: VPS34, which is connected to regulation of phagocytosis, pinocytosis, endosomal sorting and autophagy [104].

#### 3.1.2. AKT

The serine/threonine protein kinase AKT is the principal downstream molecule of the PI3K signaling pathway. There are three subtypes (isoforms) of AKT [105,106]: AKT1 (expressed in the majority of tissues), AKT2 (expressed mainly in tissues with high sensitivity to insulin: liver, pancreas, muscles), and AKT3 (expressed in the brain and testicles). AKT is activated by PIP2-driven and PIP3-driven recruitment to the plasma membrane. Here, the phosphorylation of Thr308 and of Ser473 determines the activation of AKT [107,108].

Activated AKT mediates the regulation of the cell cycle, growth, proliferation, and energy metabolism [109]. AKT has over 100 substrates [105], including: transcription factors, inhibitors of cell cycle progression, protein kinases, GTPase-activation proteins, and apoptosis inducers [110,111].

Glycogen synthase kinase-3 (GSK-3), one of the main AKT protein substrates, is a protein kinase that phosphorylates and inhibits the glycogen synthase. GSK-3 lies downstream of multiple cellular signaling pathways, such as: the phosphatidylinositol-3–kinase-dependent pathway that is stimulated by insulin and growth factors, and the Wnt signaling pathway that is required for embryonic development [112]. GSK-3 is primarily regulated by inhibition [113]. There are two isoforms, GSK-3α and GSK-3β, generated from distinct genes, but with great structural homology (almost 97%) and similar roles, being encountered in many tissues [112,113] and especially in the brain. AKT phosphorylates GSK-3 and inactivates it; consequently, there is an increase in the cellular uptake of glucose and glycogen synthesis. This determines a decrease of blood sugar levels [114].

The inhibition of GSK-3 triggered by growth factors, through AKT activation, has anti-apoptotic effects [113]. GSK-3 has a broad range of substrates (more than 100) including signaling proteins, structural proteins, and transcription factors involved in metabolism [115].

#### 3.1.3. mTOR

TOR is a large protein-kinase inactivated by a bacterial toxin called Rapamycin—hence the name Target of Rapamycin. It was identified in yeasts, but it also exists in mammalian cells, being named mTOR (mammalian Target of Rapamycin) [94,116]. In cells, it exists as two distinct multiprotein complexes: mTORC1 and mTORC2. mTORC1 contains mTOR, protein Raptor and mLST8 (the acronym for mammalian Lethal with SEC13 protein 8). mLST8 interacts directly with mTOR and enhances its kinase activity, with this protein being found in human colon and prostate cancer cells [117]. mTORC1 is sensitive to Rapamycin and promotes cell growth and survival by stimulating nutrient uptake and metabolism [94,116]. It also stimulates cell growth by promoting ribosome production and protein synthesis and by inhibiting protein degradation [94,116]. mTORC1 may be activated through different pathways, but mainly through the PI3P/AKT pathway, which is activated by extracellular growth factors and nutrients. Activated AKT phosphorylates the Tuberous Sclerosis protein 2 (TSC2), which becomes inactive. Thus, TSC2 cannot keep Rheb (a Ras-related GTPase) in its inactive form. Consequently, Rheb-GDP (inactive) becomes Rheb-GTP (active), contributing to the activation of mTORC1 [94,116,118]. Other targets of the mTORC1 are: S6K (a protein kinase that phosphorylates the ribosomal protein S6) and 4E-BP (an inhibitor of the translation initiation factor eIF4E); the consequences are increased production of ribosomes and increased protein synthesis [118].

mTORC2 consists of mTOR, protein Rictor, Sin1 and mLST1 and is not sensitive to Rapamycin. mTORC2 promotes AKT activation by directly phosphorylating its hydrophobic motif (Ser473). This permits further phosphorylation of AKT, at Thr308, by PDK1 and so AKT becomes fully active [94,116]. Sin1 contains a phospholipid-binding pleckstrin homology (PH) domain that facilitates the association of mTORC2 with membranes [119]. Phosphorylation of Sin1 at Thr86 and Thr398 (by S6K or AKT) dissociates Sin1 from mTORC2, thus resulting mTORC2 inhibition [120]. Acting on S6K, mTORC1 directly regulates mTORC2 [120]. mTORC2 is mainly involved in the reconstruction of the cytoskeleton (through the Rho family GTPases) and cell survival [94].

#### 3.1.4. FoxO1

Forkhead box other 1 is a member of the Forkhead transcription factor family. The family is divided into 17 subfamilies named FoxA to FoxQ [121]. There is a common feature of the Forkhead family, namely a conserved DNA-binding domain called Fox [122].

FoxO1 is important for the glucose and lipids’ metabolism. It enhances the synthesis of enzymes involved in gluconeogenesis, has a suppressive effect on the synthesis of enzymes of glycolysis, inhibits the pentose phosphate pathway, and diminishes the triacylglycerol synthesis. Insulin activates the RTKs and initiates the PI3P/AKT pathway. The activated AKT phosphorylates the FoxO1 existing in the cytosol. The phosphorylated FoxO1 is tagged by the attachment of ubiquitin and is then degraded by proteasomes. The unphosphorylated FoxO1 remains active, passes from the cytosol into the nucleus, binds to a response element, and triggers the transcription of its associated genes, such as PEP-carboxykinase, glucose 6-phosphatase, etc. FoxO family members have an important role in oxidative stress resistance, cell proliferation, apoptosis, and differentiation [123,124].

#### 3.1.5. PTEN

**Figure 1:**
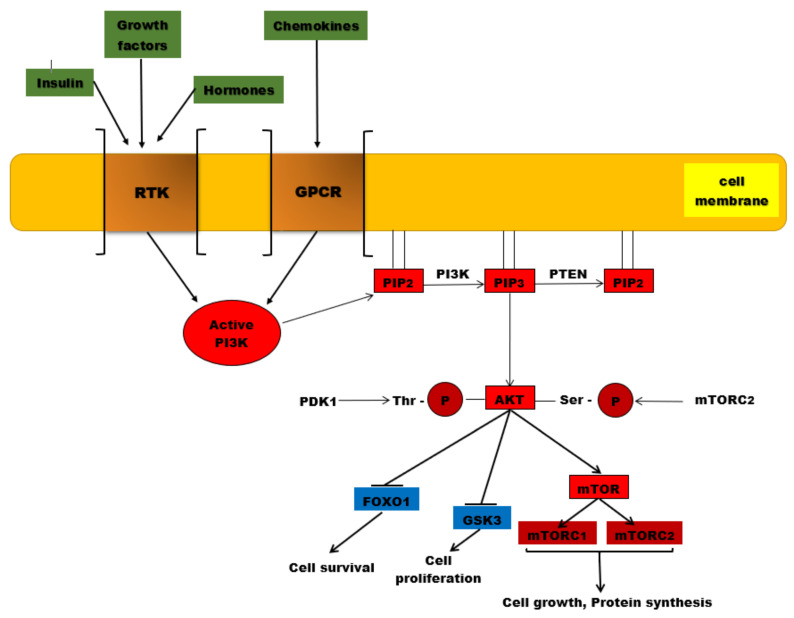
The PI3K/AKT/mTOR signaling pathway. PI3K is activated by the binding of ligands (insulin, growth factors, hormones) to RTKs, but also to GPCR (chemokines). Once activated, this protein kinase will catalyze the phosphorylation of PIP2 to PIP3. AKT is recruited to the plasma membrane where it undergoes two phosphorylation processes, one catalyzed by PDK1 at the level of threonine residue and the second reaction being catalyzed by mTORC2. Once activated by phosphorylation, AKT will phosphorylate other substances such as the mTOR complex, which will be associated in the end with protein synthesis and cell growth. Other phosphorylated substrates, such as GSK-3 and Fox01, will be inhibited, associated with cell proliferation and survival. PTEN is the major negative regulator of this signaling pathway involved in PIP3 dephosphorylation.

PTEN (phosphatase and tensin homolog) is a PIP3 specific phosphatase that dephosphorylates the PIP3 molecules, resulting in PIP2 molecules (Figure 1). PIP2 is not a binding dock for AKT, so AKT cannot be recruited to the cell membrane. As a consequence, AKT cannot be phosphorylated by mTORC2 on Ser473; therefore, the conformation of the AKT does not change anymore and the phosphorylation on Thr308 by PDK1 is not permitted. The result is that AKT cannot be activated and the PI3K/AKT/mTOR signaling pathway is suppressed [125,126]. That is why PTEN acts as a tumor suppressor, by inhibiting cell proliferation [125,126]. In many malignant tumors, the PTEN gene has suffered mutations, resulting in abnormal PTEN, which cannot exert its inhibitory effect on the PIP3/AKT/mTOR pathway [127]. The plasmatic levels of PIP3 rise and the activity of AKT is continuously stimulated [128]. By modulating the PIP3/AKT/mTOR pathway, PTEN is linked to glucose homeostasis [129].

## 4. PI3K/AKT/mTOR Mutations in Breast Cancer

The PI3K pathway undergoes many changes in breast cancer caused by mutations or amplifications of genes which encode the catalytic subunits p110α (PIK3CA) and p110 β (PIK3CB), but also the regulatory subunit PI3K, p85α (PIK3R1) [130]. In human neoplasms, PIK3CA is the frequently mutated gene that encodes the p110α catalytic subunit of the PI3K pathway, and was found amplified in head and neck, cervical, gastric, lung and breast cancers. In prostate, breast, endometrium and colon cancers, the highest incidence of PIK3CA mutations has been detected [131].

Approximately 30–40% of patients with breast cancer present PIK3CA mutations, which will induce hyperactivation of the α isoform (p110α) of PI3K. Recently, the FDA (Food and Drug Administration) approved testing of breast cancer patients with PIK3CA mutations using breast tumor tissue and/or circulating tumor DNA, isolated from plasma specimens. The results reported 11 PIK3CA hotspots mutations, located mainly in exons 9 and 20. Gene PIK3CA mutations have been detected using a PCR test, with the results revealing the following exon mutations—exon 9: E542K, E545A, E545D, E545G, E545K, Q546E, and Q546R; and exon 20: H1047L, H1047R, and H1047Y [132]. PI3Kα is activated both by binding insulin or growth factors to RTKs and by oncogenic mutations [133]. In breast cancer, the PI3K/AKT pathway is activated through PIK3CA or AKT1 mutations and PTEN loss [134].

In 2004, Samuels Y and co-workers reported somatic mutations of PIK3CA coding p110α in various solid malignancies for the first time [135]. Samuels Y et al., observed that the majority of PIK3CA somatic mutations are located at the level of exon 9 (E542K or E545K) and exon 20 (H1047R or H1047L). In the helical domain of p110α, there are exon 9 mutations that are considered to enable p110α to escape the inhibitory effect of p85 via the Src-homology 2 (SH2) domain. Near the activation loop of the kinase domain, mutations of exon 20 are located. The study reported 10% frequency of PIK3CA somatic mutations in breast cancer, but later studies reported ∼30%. [136]. Karakas B et al., also reported that the catalytic subunit of the PI3K gene called PIK3CA or p110α is frequently mutated in breast cancer [137].

In the coding sequence, PIK3CA mutations are concentrated in three hotspots, with two located in the helical domain of p110α and the last situated in the catalytic domain. The hotspot mutations represent the single nucleotide substitutions that will determine amino acid substitutions, E542 K, E545 K and H1047R. Unfortunately, these hotspot mutations induce a gain-of-function and prompt transformation and tumorigenicity. The results from 6338 tumors revealed that 2261 patients presented PIK3CA mutations (35.7%) [138].

A total of 73% of all PIK3CA mutations are: H1047R (35%), E545K (17%), E542K (11%), N345K (6%), and H1047L (4%). In patients with triple negative breast cancer, PIK3CA mutation rates were decreased (16%) compared to HR+/HER2 (42%) and HER2+ (31%) breast cancer subtypes. Moreover, in patients with advanced HR+/HER2−breast cancer, 28% of PIK3CA mutations were identified in circulating tumor DNA [132].

Tumor sequencing studies have reported that these somatic mutations of PI3CA, concentrated in certain hotspots, will lead to tumor progression by gaining a function for PI3CA [139]. Moreover, PIK3CA mutations in human breast cancers, at E545K in exon 9 and H1047R in exon 20, have been reported even by studies using cell lines such as MCF10A immortalized breast epithelial cells. PIK3CA was the most frequent mutation observed, associated with an increased kinase activity of the PI3K pathway. Mutant PIK3CA promotes cell growth and invasion of human cancer cells [136].

Bachman KE and co-workers reported an incidence of 25% PIKCA mutation in human breast cancer. The study did not reveal any correlation between PIK3CA and the presence or absence of ER/PR labelling, or even with Her-2/neu. PI3CA mutations affect the PI3K/AKT/mTOR signaling pathway independent of ER/PR and Her-2/neu. Analyzing the fifty-three samples, the study reported three mutations in exon 9, 8 uncovered mutations in exon 20, and novel somatic mutations were detected—two in exon 1 and one in exon 2 [140]. Stemke-Hale K and colleagues analyzed 547 breast tumor samples and 41 cell lines using mass spectrometry sequencing and reverse-phase protein arrays to detect mutations in PI3KCA, AKT and PTEN. The study revealed that the most common PIK3CA mutations were found in hormone receptor-positive forms (34.5%) followed by HER2- positive cases with an incidence of 22.7%, compared with basal-like tumors (8.3%). Moreover, in hormone receptor-positive cancers, mutations on AKT1 represented 1.4% and PTEN 2.3%, respectively [141]. Using cell cultures, the study reported that AKT1 mutations were absent, while PIK3CA and PTEN mutations appeared in 39% and 20% of the cases, respectively. In tumors and cell lines, PIK3CA mutations compared with the loss of PTEN and AKT1 mutations were associated with less activation of AKT. The most frequent modifications on the PI3K/AKT/mTOR signaling pathway were PTEN loss and PIK3CA mutation [141].

Li SY. et al., analyzed 250 primary human breast tumors and detected that 35% of PIK3CA mutations were located in C2 helical and kinase domains. The PIK3CA mutations were associated with larger tumors and significantly worse survival rate, especially in positive estrogen receptor status or non-amplified ERBB2 [142]. Moreover, PIK3CA mutations may sometimes harbor PTEN loss or HER2 overexpression in breast tumors [130].

p110α, the catalytic subunit of the phosphoinositide 3-kinase alpha (PI3Kα) complex, which is necessary for normal growth and proliferation, [133] is essential for signaling and the growth of tumors driven by PIK3CA mutations or RTKs [130]. It has been shown that p110β mediates tumorigenesis in PTEN-deficient cells [139]. Breast cancers show poor disease outcome if they are associated with increased levels of AKT phosphorylation/activation and PTEN loss. Moreover, the loss of PTEN activity and activation of the PI3K signaling pathway are associated with resistance to endocrine therapy [134]. Endometrial, prostate, breast, thyroid and kidney tumors present somatic PTEN alterations, leading to uncontrolled PI3K activation [143]. PTEN, the most important regulator of the PI3K/AKT/mTOR signaling pathway, is involved in cell growth and survival, cellular migration and genomic stability. In 1997, it was discovered that PTEN acts as a key tumor suppressor gene for various tumor types, being involved in cell cycle progression, cell growth and survival. Moreover, PTEN is implicated in DNA repair and genome stability. In response to DNA damage, PTEN is phosphorylated (Tyr) and binds to chromatin, promoting DNA repair [144].

The somatic mutations (missense and nonsense mutations, monoallelic or biallelic deletion on the PTEN gene), epigenetic alterations (methylation promotor), PTEN protein degradation and the post-translational modification of PTEN protein will conduce to PTEN inactivation. In breast tumors, the loss of heterozygosity at the PTEN locus was detected in 40–50% cases. The loss of PTEN function due to PTEN mutations is found in 5–10% of breast cancers [144].

In luminal breast cancers, the PI3K pathway is one of the most altered pathways, correlated with PIK3CA mutations, loss of PTEN, or downstream protein phosphorylation [145]. Zardavas D et al., reported the results obtained from 10,319 patients included in 19 studies where PIK3CA mutations were present in 32% of patients. PIK3CA mutations were associated with ER positivity, and were increased with age, lower grade, and smaller size. In breast cancer subtypes-ER-negative/HER2-negative, HER2-positive, and ER-positive/HER2-negative, the prevalence of PIK3CA mutations was 18%, 22%, and 37%, respectively [146]. Ling D et al., conducted a study in which tumors from 507 breast cancer patients were collected from the West China Hospital between 2008 and 2013. The study’s results revealed 3.% AKT1 mutations with ER+/PR+/HER2. The incidence of the PIK3CA mutations was reported at 46.5%. These mutations were associated with ER+/PR+/HER2‒ status, and it was observed that 35 patients carried two or three variants of the PIK3CA gene [147]. PIK3CA mutations, associated with many distinct cancers, include hotspot single–amino acid substitutions in the helical (E542K and E545K) or kinase (H1047R) domains. In multiple cancer types, including breast cancer, PIK3CA is considered oncogenic, mutations of the alpha catalytic subunit of PI3K having an incidence of 40% in ER+/HER2− primary and metastatic tumors. Therefore, PIK3CA is a target for cancer therapy [134]. Anderson EJ et al., reported 36% PIK3CA mutation in HR+/HER2- metastatic breast cancer [148].

Mutations also occur in RTKs such as HER2 (ERBB2) and fibroblast growth factor receptor (FGFR)1, in AKT1, AKT2, PDK1, and loss of PTEN and INPP4B (inositol polyphosphate-4-phosphatase type II). The activation of PI3K occurs through the binding of growth factors to RTKs and GPCR. Moreover, PIK3CA mutations appear in breast tumors associated with PTEN loss or HER overexpression [130].

Lehmann BD and co-workers detected highly clonal PIK3CA mutations in the triple negative breast cancer subtype that present a luminal phenotype and express androgen receptors (40%) versus triple negative breast cancer without androgen receptors (4%) [149]. A total of 15–20% of breast cancer cases present an overexpression of human epidermal growth factor receptor-2 (HER2), associated with an aggressive clinical behavior [150]. Luminal A tumors are associated with PIK3CA mutations in 45% of the cases, while AKT1 and PTEN mutations both appear in 4% of the patients. At the same time, PIK3CA genes are mutated in 29% of the cases with subtype luminal B, in 39% of HER2-enriched breast cancers and only in 7% of basal-like tumors [151].

### HER Receptors and Breast Cancers

EGFR (epidermal growth factor receptor, also known as ERBB1/HER1), ERBB2 (HER2), ERBB3 (HER3), and ERBB4 (HER4) represent the ERBB family of RTKs, which are cytoplasmic membrane-anchored proteins. All four receptors display similarities in structure and sequence, contain an extracellular ligand-binding domain, a transmembrane domain, and an intracellular tyrosine kinase domain [152].

Cell growth, survival, and differentiation are regulated by HER receptors via various signaling pathways and even participate in cellular proliferation and differentiation. The HER2 gene encodes a 185-kDa transmembrane protein, being located on the long arm of chromosome 17 [153]. When HER2 is overexpressed or amplified, it stimulates tumor growth, invasiveness, and survival via the activation of several signaling cascades, such as PI3K/AKT pathways. HER2 phosphorylation may lead to PI3K/AKT/mTOR pathway activation [154].

The formation of HER2-EGFR dimers, HER2 homodimers and even HER2-HER3 dimers will promote tumor development by increasing tumor cell metabolic functions, cell survival, proliferation and invasiveness [155]. In breast cancer, overactivation of HER receptors is caused by several factors such as gene amplification, truncation of the extracellular domain, mutations in the kinase domain, and co-expression of HER receptor ligands [156]. HER2 overexpression is associated with poor clinical outcome and disease progression [152].

In primary invasive breast cancer, approximately 18–20% of cases present an amplification or overexpression of the HER2 oncogene [154]. HER 2 (c-erbB-2) is a cell membrane surface-bound RTK, while HER2/neu, its extracellular domain, is normally implicated in the signal transduction pathways that will conduce to cell growth and differentiation. In approximately 15–20% of breast cancer cases, HER2-overexpression was observed [157]. HER2 overexpression and PIK3CA mutations have been observed in both invasive breast cancers and ductal carcinoma in situ. In intraepithelial neoplastic lesions, PIK3CA mutations have a decreased frequency, so these mutations can enhance PI3K pathway activation by HER 2 (ERBB2) [130].

EGFR, HER3, and HER4 are amplified and overexpressed in more than 20% of breast cancers. Moreover, HER2 is the oncogenic driver of these pathologies, involved in the genesis and progression of these tumors [158]. EGFR and HER4 can activate PI3K after their binding to RTKs, especially by transphosphorylation of HER3, which can act as a critical partner for HER2 in the genesis and progression of the tumor [158]. After phosphorylation of tyrosine residues within the cytoplasmic domain, dimerization of the receptor takes place and various signaling pathways are activated, which are further involved in cellular proliferation, transcription, motility, and inhibition of apoptosis [154].

Yang Z et al., conducted a study that included 142 patients with metastatic breast cancer, detecting alterations in estrogen receptor (ER), progesterone receptor (PR), and HER2 status as follows: 20.70%, 37.78%, and 11.48%, respectively [159].

## 5. Mechanisms of Endocrine Resistance in Breast Cancer

Breast cancer, the most common form of cancer in women, has a very high mortality rate, causing the death of a woman every 13 minutes. One of the most important factors involved in breast cancer development and progression is represented by the expression of proteins for hormone receptors, with estrogen positive breast cancers representing seventy percent of the total cases. Therefore, endocrine therapy plays a crucial role in breast cancer therapy [160,161,162,163,164,165].

HER2+ breast cancers account for 15% to 20% of all cases and are treated primarily with drugs that target HER2 (trastuzumab, pertuzumab). In addition, half of these cases are also ER positive; therefore, these women are perfect candidates for endocrine therapy, despite the shorter and lower response rate compared to HER2 negative breast cancers [166].

However, de novo and acquired resistance appear in all cases of metastatic breast cancer and in approximately 25% of ER positive breast cancer patients, limiting the efficiency of the treatment used to target the estrogen receptor [162,166]. Although the exact mechanisms that lead to endocrine resistance have not been identified, there are several theories revealing cell cycle changes and alterations of the ER pathway as causes for endocrine resistance [166]. Moreover, studies have shown that growth factor receptor signaling pathways are involved in the development of this aggressive pathology. In addition, PI3K mutations or loss of heterozygosity, methylation of PTEN, and AKT activation promote hormonal therapy resistance, thus the new treatment protocols are based on the use of medicines targeting not only the estrogen receptor but also these signaling pathways [162,167].

Previous studies have shown that ligand-independent estrogen receptor activity caused by mutations in the encoding gene for ER (ESR1) can lead to endocrine resistance through an increased number of mutant clones [166].

Estrogen positive breast cancers are currently treated with three types of agents: selective estrogen receptor modulators (tamoxifen), estrogen synthesis inhibitors (aromatase inhibitors) and selective estrogen receptor down-regulators (fulvestrant) [167].

The main goal of endocrine targeted therapy is to remove the endogenous activating ligands of the estrogen receptors. Tamoxifen and other antiestrogens (such as fulvestrant) fulfil their role through competitive inhibition, while aromatase inhibitors (letrozole, anastrozole) block estrogen synthesis [166,168].

Tamoxifen was the first therapeutic agent targeting cancer on a molecular level, showing great results in women with breast cancer, especially in estrogen receptor-positive premenopausal women. Although tamoxifen has proven to be a very efficient drug in preventing recurrence, the estrogen receptor-positive subtype remains the most aggressive type of breast cancer [160]. Tamoxifen is an antiestrogen that performs as a partial agonist and has been the standard of care for premenopausal women for many decades. However, aromatase inhibitors have been proven to increase the survival rate for postmenopausal women and have replaced tamoxifen as the main therapy [166].

Previous studies indicate that estrogen receptor breast cancers have a low recurrence rate, but the risk increases over 3 to 5 years after the initial treatment. This late recurrence, called dormancy, is often associated with ER positive breast cancers and could be determined by the therapeutic agents used to treat the disease [166].

HER2 has been incriminated in many important pathways involved in tamoxifen resistance, with an increased expression of HER2 being associated with resistance to hormonal therapy [169]. In this case, tamoxifen can perform both as an agonist or an antagonist, depending on the recruitment of coactivators or repressors of the estrogen receptor α transcription complex. In the presence of HER2, the augmented expression of AIB1 (amplified in breast cancer 1 protein), a regulator of the estrogen receptor α, leads to tamoxifen resistance [170].

Moreover, growth factor receptors such as IGF1R (insulin-like growth factor receptor 1) and EGFR (epidermal growth factor receptor) can cause a lack of response to tamoxifen by activating the MAPK (mitogen-activated protein kinase) and the PI3K signaling pathway [170,171].

The cross-talk between these receptors and ER is very complex. MAPK leads to estrogen-independent phosphorylation, with AKT playing an essential role for ERα [171]. Activation of the PI3K signaling pathway and AKT phosphorylation promotes estrogen-independent growth in tumor cells and resistance to anti-estrogens. The overexpression of HER2, FGFR1 or loss of INPP4B (inositol polyphosphate-4-phosphatase type II) was also observed in tamoxifen-resistant cells. PI3KCA (the alpha catalytic subunit of PI3K) is more often affected in estrogen positive breast cancers, while ER negative breast cancers are characterized by PTEN loss [161].

Additionally, it has been demonstrated that tamoxifen dysregulated metabolism (caused by cytochrome P450 proteins’ polymorphism), cellular accumulation of the drug, hypermethylation of CpG islands and expression of P-glycoprotein, and the activity of the histone deacetylase promote tamoxifen resistance as well. Mechanisms of aromatase inhibitor resistance implicate the PI3K pathway, MAPK, HER2 and the estrogen receptor [170].

Recent studies have shown that microRNA can promote unresponsiveness to endocrine therapy, hence new therapies could be developed to target these small RNA molecules [170]. Hoppe R et al., have discovered that miR-126 and miR-10a are overexpressed in estrogen receptor-positive breast cancers. Moreover, they found a correlation between this overexpression and the amount of time without recurrences. However, mi-R221 and miR-222 are associated with tamoxifen resistance through the decrease in estrogen receptor protein expression in tumor cells [172]. Moreover, miR-451, a microRNA that acts on PI3K/AKT and controls P-glycoprotein, presents a decreased expression in MCF-7 tamoxifen-resistant and doxorubicin-resistant cells [173].

Moreover, epithelial to mesenchymal transition is coded by genes that are active during embryogenesis tissue formation and wound healing, but also during carcinoma progression. Transcription factors (FOXC2, ZEB1/TCF8, E12/E47 and many others) determine abnormal survival via PDGFR (platelet derived growth factor), FGFR (fibroblast growth factor receptor) and EGFR, but also through PI3K, AKT and mTOR [174].

In order to improve breast cancer prognosis, new therapeutic strategies have to use combinations of drugs targeting ER and HER2, but also the downstream signaling pathways [167].

The combination of tamoxifen with PI3K inhibitors augments the effects of the antiestrogens, thus reflecting the influence of the PI3K/AKT pathway in acquired endocrine resistance [161]. In addition, studies have shown that the association of PI3K/mTOR inhibitors (BEZ235) improved the outcome of the treatment, showing better results than when the drugs were administered individually [175]. Moreover, Cavazzoni A et al., found that the addition of everolimus, an mTORC1 inhibitor, improved the effects of letrozole [176]. On the other hand, PI3K inhibitors are in the initial phase of development [177].

## 6. mTOR Inhibitors: Everolimus and Temsirolimus

The PAM (phosphoinositide 3 kinase (PI3K)/AKT/mammalian target of rapamycin (mTOR)) pathway is often altered in cancers, being involved in more than 70% of breast cancer cases. Studies show that PIK3CA2 is the most commonly mutated oncogene in ER positive breast cancers (in 35% of the clinical cases) and is frequently involved in HER positive forms of breast cancer. In addition, several studies reflect a correlation between the activation of the PAM pathway and resistance to endocrine therapy. Therefore, an increased number of clinical trials have focused on inhibitors of this key signaling pathway that is involved in essential cellular processes, such as proliferation and metabolism, being imperative for cellular survival. Although the studies have shown great perspectives for a series of PI3K and AKT inhibitors, the adverse effects of these compounds have led to the limitation of clinical trials to only one mTOR inhibitor: everolimus [178].

Everolimus is an oral rapalog (rapamycin analog) approved by the FDA as an antitumor agent in ER positive/HER negative breast cancer [178,179]. RAD-001 (40-O-(2-hydroxyethyl)–rapamycin), better known as everolimus, exerts its effects on mTOR by binding cyclophilin FKBP-12 associated with raptor and mLST8, thus acting as an inhibitor for downstream signaling [179,180].

mTOR is downstream of PI3K/AKT and consists of 2 complexes (mTORC1 and mTORC2) that function differently despite their similar structure. mTORC1 promotes mRNA translocation, protein synthesis and lipid synthesis, thus stimulating cell growth, while mTORC2 is involved in AKT phosphorylation and cellular organization. Rapalogs, just as rapamycin, target mTORC1 and can produce the phosphorylation of the activation function domain 1 of the estrogen receptor via substrate ribosomal S6K1 (S6 kinase-1), hence leading to the activation of the ligand-independent receptor [179].

In addition, mTORC1 helps mRNA translation by producing the dissociation of 4E-BP1 from eIF4E through phosphorylation and causing the formation of a pre-initiation translation complex by associating eIF4E with eIF4G (a scaffolding protein) and initiation factors. Thus, in stress conditions, mTORC1 represents a restriction point in the cells and is a key target for cancer therapy [178].

**Figure 2:**
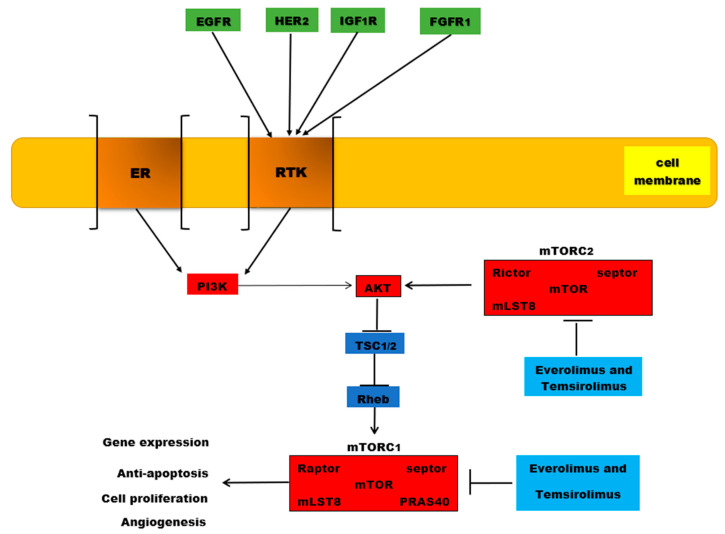
The PI3K/AKT/mTOR signaling pathway and breast cancer. The PI3K/AKT/mTOR signaling pathway is activated by ER, but also by EGFR, HER 2, IGFR1R, and FGFR1 at RTKs level. Once activated, protein kinase B or AKT inhibits TSC ½ by phosphorylation, further leading to the inhibition of Rheb and activation of mTORC1. This activation is associated with anti-apoptotic effects, increased gene expression, cell proliferation and angiogenesis. Everolimus and temsirolimus are two analogues of rapamycin that inhibit the activity of mTOR, especially mTORC1, but also mTORC2.

Interestingly, AKT can be hyperphosphorylated by rapalogs. mTORC1 inhibition and inadequate inhibition produce activation of AKT and cause cell proliferation [178,179]. The pharmaceutical properties of everolimus are superior to rapamycin. This hydroxyethyl ether derivative of rapamycin does not inhibit mTOR at pharmacologically achievable drug concentrations [179]. Rapalogs’ influence on mTORC2 is very controversial. While some specialists consider that everolimus can exert its effects on both mTOR complexes, most authors consider that this agent can only act on mTORC1 (Figure 2) [181].

Bachelot et al., analyzing the effects of tamoxifen and of tamoxifen combined with everolimus, showed that the clinical benefit rate and time to progression were significantly improved by the rapalog [182].

Balsega J. et al., evaluated 724 patients in the BOLERO (Breast Cancer Trial of Oral Everolimus)-2 study, assessing the effects of everolimus and exemestane (an aromatase inhibitor) in postmenopausal women with advanced stages of hormone positive receptor breast cancer. Their results showed that everolimus improved the progression free survival rate. However, the combination of the two therapeutic agents also showed adverse effects, such as anaemia, pneumonitis or dyspnea, causing the withdrawal of everolimus [180].

The BOLERO-3 study evaluated the effect of everolimus in HER2 positive, trastuzumab-resistant breast cancer. This randomized study Phase III trial included 569 women with advanced breast cancer and resistance to HER-targeted drugs. The results showed that the addition of everolimus to a combination of trastuzumab and vinorelbine (a chemotherapy medication) significantly improved the progression free survival of the patients [183]. Additionally, everolimus showed great promise in association with trastuzumab and paclitaxel in HER2-positive advanced breast cancers [184].

The MANTA trial (phase 2 randomized clinical trial) is a study that included 333 women with ER positive breast cancer, priorly treated with aromatase inhibitors. The participants were divided into 4 categories depending on the treatment they received: group 1 comprised of 67 women that received fulvestrant, the second group was represented by 103 patients that received fulvestrant and vistusertib (a dual mTORC1 and mTORC2 inhibitor) every day, the third group comprised of 98 patients that intermittently received the combination of fulvestrant and vistusertib, while the fourth group was formed by 65 patients that received fulvestrant and everolimus. This study was conducted in 9 countries and it demonstrated that everolimus in combination with fulvestrant significantly improved progression free survival, thus also reflecting the superior therapeutic effect of rapalogs over mTOR dual inhibitors [181].

Moreover, studies have shown that everolimus enhances letrozole effects, blocking the breast cell cycle and stimulating apoptosis [185]. On the other hand, another study that included 120 women with hormone receptor-positive metastatic breast cancer treated with endocrine therapy and more chemotherapy agents revealed that no significant benefits were obtained by adding everolimus to the therapeutic strategy [186].

Temsirolimus (CCI-779), another rapamycin analog, is converted into rapamycin in vivo [133]. Yu et al., highlighted the sensitivity of inhibitor CCI-779 to MCF-7 cells, determined by the amplification of a kinase (p70S6) downstream of AKT regulated by mTOR [187]. The HORIZON trial, a randomized study, showed that temsirolimus in combination with letrozole did not improve the progression free survival of patients with metastatic hormone receptor-positive breast cancer compared to letrozole alone [188]. Similar results were observed by Fleming et al., who studied the effects of temsirolimus in women with advanced breast cancer and found no significant improvement in the progression free survival rate [189] (Table 1). However, a study conducted by Sadler showed that when used in combination with ERA−923, an ER antagonist, temsirolimus displays promising results in ER positive breast cancers [190].

Dual mTOR inhibitors manifest antitumoral activity through the inhibition of mTORC1 and mTORC2, thus representing a promising strategy for breast cancer treatment. However, while the first generation of inhibitors acting on mTORC1 has been approved in clinical trials, dual inhibitors are still tested on cell cultures and animal models.

Vistusertib (AZD2014) is a dual inhibitor of both mTORC1 and mTORC2 that, compared with everolimus, has demonstrated more complete growth inhibition and cell death both in vitro and in vivo, based on a greater inhibitory function against mTORC1 and additional inhibition of mTORC2, especially in ER-positive breast cancer models. In preclinical models, vistusertib induces rapid tumor regression [181]. Bhattacharyya GS et al., conducted a Phase I/II trial that included 400 patients divided into two groups, diagnosed with hormone receptor-positive and HER2-negative breast cancer, who received tamoxifen and sirolimus 2 mg daily. The results of the study revealed that this combination of sirolimus and tamoxifen was effective and well tolerated by breast cancer patients [191]. In 2015, Seiler M and co-workers published the results of the phase IIb trial that consisted of patients with human epidermal growth factor receptor 2–positive (HER2+) trastuzumab-refractory metastatic breast cancer, who received a daily dose of ridaforolimus and trastuzumab. The study observed that the combination ridaforolimus–trastuzumab was well tolerated. Moreover, in trastuzumab-resistant HER2 positive metastatic breast cancer patients, this combination has antitumor activity [192].

Furthermore, the second-generation mTOR inhibitors are able to inhibit the kinase activity of both mTORC1 and mTORC2, having a more potent anticancer activity compared to rapalogs. In vivo and in vitro experiments revealed that AZD8055 is an mTOR kinase inhibitor with antitumor activity. Moreover, AZD8055 can treat breast cancer resistance to endocrine therapy agents, such as tamoxifen and fulvestrant. Shi JJ et al., detected in breast cancer cells that AZD8055 may overcome tamoxifen resistance [193]. Jordan NJ et al., used in vitro breast cancer cells (MCF7-X) that were treated with everolimus (RAD001) or AZD8055 alone or combined with anti-hormone fulvestrant. RAD001 presented a poor growth inhibitory effect on cells, rapidly inhibiting mTORC1 but not mTORC2. In contrast, AZD8055 rapidly inhibited both mTORC1 and mTORC2, and displayed a powerful inhibitory effect on cells’ growth [194]. So far, another dual mTOR inhibitor, MLN0128 is used only in vitro on cell models against everolimus-resistant breast cancer, and it inhibits the AKT phosphorylation. MLN0128 may suppress the proliferation of this kind of cell [195]. Bostner J and co-workers detected that raptor protein expression in the nucleus was increased in ER/PgR-positive and HER2-negative tumors with low grade, further associated with the luminal A subtype. Moreover, raptor seems to stimulate the growth of the luminal A subtype and may be a possible target along with endocrine treatment [196]. Zhu L et al., treated human breast cancer cell lines (MCF-7 and ZR-75–1) with tamoxifen or rapamycin, to observe if ER positive breast cancer cell growth is inhibited. It was observed that rapamycin enhanced the effects of endocrine therapy with tamoxifen. In vivo treatment of cells with rapamycin plus tamoxifen significantly inhibited tumor growth [197]. mTOR is involved in PI3K/AKT signaling pathways, being associated with cell survival, proliferation, metabolism, and angiogenesis, and being abnormally activated in breast cancer. mTOR inhibitors have been developed to enhance the antitumor activity through complete mTORC1 inhibition and mTORC2, which promotes AKT activation by phosphorylation.

Corroborated, these results exhibit the key role played by mTOR inhibitors, especially by everolimus, in the treatment of ER positive/HER2 positive breast cancers with endocrine resistance.

## 7. Conclusions

Unfortunately, breast cancer has a high risk of mortality among women, with seventy percent of estrogen positive cases; therefore, endocrine therapy is crucial for this kind of neoplasia. Many factors are involved in breast cancer pathologies—some of them are risk factors, others are considered to be protective factors or can be without any influence on breast cancer.

The PI3K/AKT/mTOR signaling pathway is activated by enzyme-linked receptors and is of paramount importance in cell differentiation, proliferation, energetic and glucose metabolism, apoptosis, cellular response to oxidative stress, and angiogenesis. In breast cancer, the PI3K pathway presents mutations of genes which encode the catalytic and the regulatory subunits. The most frequent mutations are located in exon 9 and 20, identified from tumor tissue and/or circulating DNA in all breast cancer subtypes. Breast cancer mutations also appear in RTKs receptors such as HER 2, and phosphorylation of this receptor leads to PI3K/AKT/mTOR activation.

PI3K mutations, PTEN methylation and AKT activation will result in hormonal therapy resistance. Everolimus is a rapamycin analog approved by the FDA which inhibits the mTOR complex, involved in mRNA translocation, protein and lipid synthesis, promoting cell growth and cellular organization. Regarding another rapamycin analog, temsirolimus, the clinical studies conducted so far have reported promising results in ER positive breast cancers.

In conclusion, the investigation of mutations that occur in the PI3K/AKT/mTOR signaling pathway, but also of its inhibitors, may be a real benefit for patients diagnosed with breast cancer. By inhibiting cell growth and proliferation, these drugs could play an essential role in malignant cells’ death. Therefore, future studies should focus on reducing the side effects of dual inhibitors in order to optimize the efficiency of these drugs. Moreover, new combinations of the inhibitors of these crucial pathways involved in the development of breast carcinoma could provide a new perspective for the management of breast cancers, especially for the cases with resistance to endocrine therapy. Furthermore, genetic profiling of the patients could lead to a better case selection in which PAM inhibitors would prove to be the key therapeutic agents, particularly for patients with poor prognosis.

## Figures and Tables

**Table 1:** The main mTOR inhibitors, used in various clinical trials in patients with different breast cancer types.

mTOR Inhibitors	Type of Breast Cancer	Type of Study	References
**Everolimus + exemestane**	hormone-receptor-positive advanced breast cancer	Phase 3, randomized trial	[180]
**Everolimus + fulvestrant**	estrogen receptor-positive breast cancer	Phase 2 Manta trial	[181]
**Everolimus + tamoxifen**	metastatic breast cancer	Phase II Randomized trial	[182]
**Everolimus + plustrastuzumab + vinorelbine**	HER2-positive breast cancer	Phase 3 trial (Bolero-3)	[183]
**Everolimus + trastuzumab + paclitaxel**	HER2-positive advanced breast cancer	Phase 2 multicenter study	[184]
**Everolimus**	metastatic breast cancer	Retrospective study	[186]
**Temsirolimus + letrozole**	hormone receptor-positive metastatic breast cancer	Phase III randomized trial	[188]
**Temsirolimus**	metastatic breast cancer	Phase II trial	[189]
**Sirolimus + Tamoxifen**	hormone receptor-positive and HER2-negative breast cancer	Phase I/II trial	[191]
**Ridaforolimus + trastuzumab**	Human epidermal growth factor receptor 2–positive (HER2+) trastuzumab-refractory metastatic breast cancer	Phase IIb trail	[192]
